# Nested Named Entity Recognition using Multilayer BERT-based Model: Notebook for the BioASQ Lab at CLEF 2024

**Published:** 2024-09

**Authors:** Hasin Rehana, Benu Bansal, Nur Bengisu Çam, Jie Zheng, Yongqun He, Arzucan Özgür, Junguk Hur

**Affiliations:** 1 School of Electrical Engineering & Computer Science, University of North Dakota, Grand Forks, North Dakota, 58202, USA; 2 Department of Biomedical Sciences, School of Medicine and Health Sciences, University of North Dakota, Grand Forks, North Dakota, 58202, USA; 3 Department of Biomedical Engineering, University of North Dakota, Grand Forks, North Dakota, 58202, USA; 4 Department of Computer Engineering, Bogazici University, Istanbul, 34342, Turkey; 5 Unit for Laboratory Animal Medicine, Department of Microbiology and Immunology, University of Michigan, Ann Arbor, Michigan, 48109, USA; 6 Center for Computational Medicine and Bioinformatics, University of Michigan, Ann Arbor, Michigan, 48109, USA

**Keywords:** Named entity recognition (NER), Nested NER, Bidirectional encoder representation transformer (BERT), Natural language processing (NLP)

## Abstract

In natural language processing, named entity recognition (NER) is a crucial task involving finding and categorizing text entities. The biomedical domain presents substantial hurdles due to the complex structure of the language and the existence of nested entities. This paper introduces an innovative method for Nested NER by utilizing a multilayer bidirectional encoder representation transformer (BERT)-based model, notably employing pretrained PubMedBERT. Our proposed model is designed to manage nested entities’ complexities effectively. We combined the robust contextual embeddings from PubMedBERT with a multilayer tagging process. This approach allowed the model to precisely differentiate between overlapping items, a frequent occurrence in biomedical literature. To assess the effectiveness of our Multilayer NER Model (MultilayerNERModel), we conducted thorough experiments on the BioNNE English Dataset, a dataset for a shared task of BioASQ competition. The findings suggest that employing a multilayer approach enhances the model’s ability to identify nested entities, resulting in the thorough detection of entities in biomedical texts. It earned the highest overall performance in English oriented track, with an F1 score of 67.30% and a macro F1 score of 56.36%. These results demonstrate the significant impact of utilizing a multilayer approach in Nested NER tasks, especially in the biomedical domain. The use of UMLS dictionaries, along with the MultilayerNERModel, further enhances the model’s performance in biomedical entity recognition.

## 1. Introduction

Identifying and classifying entities, including but not limited to medical terms, names of people, organizations, and locations, is a critical undertaking in NLP. Nested NER extends this challenge by requiring the identification of entities embedded within other entities, adding complexity, particularly in specialized domains such as biomedicine [1]. In biomedical text mining, accurate Nested NER systems are essential for extracting meaningful information from scientific literature, which is crucial for advancing research and clinical practice [2]. The biomedical nested named entity recognition (BioNNE) task [3] was introduced to address this need as part of the BioASQ Workshop at CLEF 2024 [4]. This task focuses on developing and evaluating NER systems capable of handling nested entities within biomedical texts. The BioNNE task includes three tracks: English, Russian, and Bilingual. Our participation was primarily in Track 2 - English-track, though we also applied our approach to the other tracks. This English track required participants to develop a Nested NER model for English biomedical scientific abstracts. Participants could train any model architecture on any data provided by organizers to achieve the best performance, fostering innovation and applying diverse methods.

Nested NER has been thoroughly investigated in the domain of NLP while biomedical Nested NER aims to identify entities such as proteins, genes, diseases, and drugs within biomedical literature [5]. Traditional methods, such as rule-based approaches and early machine learning models, have been gradually substituted with advanced techniques that employ deep learning and pretrained language models. One of the pioneering works in biomedical NER is the introduction of BioBERT [6], a variant of the BERT model, specifically pretrained on biomedical literature from PubMed [7, 8]. BioBERT demonstrated significant improvements over previous models in various biomedical NER tasks, highlighting the effectiveness of domain-specific pre-training [9]. PubMedBERT was developed as a domain-specific model following the success of BioBERT [10]. It was trained purely on PubMed abstracts and designed to enhance the precision and effectiveness of biological NLP activities by utilizing a larger and more targeted dataset.

Nested NER addresses identifying entities embedded within other entities, which is a frequent occurrence in biomedical texts [11]. Conventional flat NER models lack the necessary capabilities to handle such intricacies. Various methods have been suggested to address the issue of Nested NER, such as layered models, span-based models, and sequence-to-sequence models [12].

**Figure 1:**
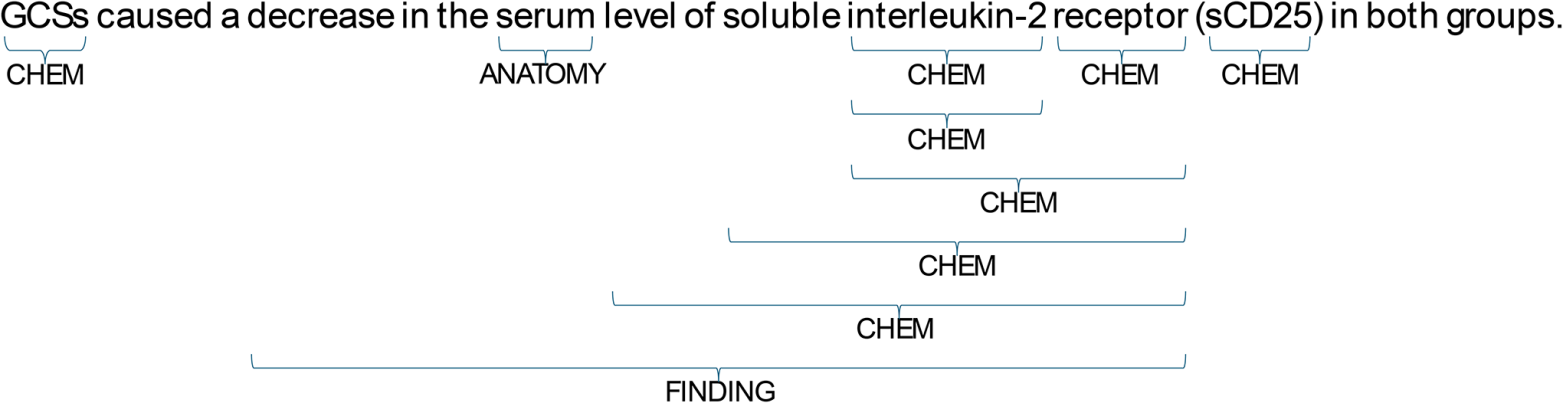
A sentence from the BioASQ-BioNNE 2024 dataset containing nested entities.

Figure 1 presents a sentence extracted from the BioASQ-BioNNE 2024 dataset, illustrating the concept of nested named entities within a biomedical context. It highlights how chemical entities like “interleukin”, anatomical entities like “serum” and finding entities such as “decrease in serum level of soluble interleukin-2 receptor” can be nested within each other, showcasing the complexity of biomedical text that advanced NER systems must handle. To participate in this challenge, we utilized PubMedBERT, a language model pretrained on PubMed abstracts, which is particularly suited for biomedical text processing [10]. By leveraging PubMedBERT, we aimed to enhance the performance of our NER system in recognizing and classifying nested entities in the complex domain of biomedical literature.

## 2. Related Work

MMBERT is a transformer-based model designed to improve the performance of biomedical NER by integrating multiple models [13]. It also uses the ERNIE-Health, a Chinese pretrained biomedical language model. While evaluating MMBERT, they used Chinese biomedical NER datasets. Other than BERT-based encoder models, GPT-based decoder models are also used for the biomedical domain. BioGPT [14] is a GPT-2-based biomedical language model that was pretrained on a large amount of PubMed articles. BioGPT has been evaluated on six different tasks. Another study investigates the benefits of fine-tuning GPT-3 for biomedical tasks such as NER, relation extraction, and question answering [15]. Their experiments on BC5CDR [16], CADEC [17] and ADE [18] showed that their fine-tuned GPT-3 models lagged behind the state-of-the-art models.

Some studies have worked on creating datasets designed explicitly for NER tasks involving biomedical entities. NEREL-BIO [19] is a detailed dataset focusing on nested named entities in the biomedical domain. It comprises over 700 Russian and 100 English PubMed abstracts, annotated to capture complex biomedical information through nested entities. The NEREL-BIO has 17 specific biomedical entity types. The dataset was created as an extension of the general-domain NEREL dataset [20]; therefore, it is an excellent resource for cross-domain and cross-language benchmarks. Their experiments with BERT-based and sequence-based models showed that the performance depends on the type of the NER.

Others introduced a novel bi-encoder framework to improve NER through contrastive learning [21]. Rather than treating NER as sequence labeling or span classification, the bi-encoder framework represents the problem as learning vector representations. By mapping candidate text spans and entity types into the same vector space, the model maximizes the similarity between an entity mentioned and its type while minimizing the similarity of the non-entity types.

## 3. Methodology

### 3.1. Dataset

To evaluate the efficacy of our proposed method, we performed experiments using the BioASQ-BioNNE dataset, which was released in 2024 as a dataset for the shared task challenge of BioASQ [22, 23, 24]. This dataset includes 54 training abstracts, 50 development abstracts, and 500 testing abstracts from PubMed for the English track. The challenge organizers provided 716 training abstracts, 50 development abstracts, and 500 testing abstracts for the Russian track. The Bilingual track participants should use the dataset provided for both English and Russian. The dataset encompasses a total of eight different biomedical named entity classes. For this article, we mainly focused on the English track. Table 1 shows the detailed distribution of different entity types across the training and development sets of the dataset. “DISO” and “ANATOMY” entities are the most frequent term classes, indicating a focus on anatomical and disorder related information, whereas “DEVICE” entity is the least frequent, suggesting limited data on medical devices. Since the annotation files for the testing data have not been disclosed to participants, we do not have the class-wise distribution for the testing set.

Each abstract in the original dataset is accompanied by a corresponding annotation file. We processed the dataset by splitting each abstract into sentences and mapping the corresponding annotations to these sentences. We implemented the BIO-tagging scheme, a well-known method for named entity recognition encoding. Tokens were encoded as “B-TYPE” for the beginning of an entity, “I-TYPE” for subsequent tokens of the same entity, and “O” for tokens that do not belong to any entity class. Given that the provided annotation files indicate up to six nested levels, we applied six levels of BIO-tagging.

After processing, we shuffled the sentences and split the merged dataset into training and validation sets using an 80:20 ratio. After performing hyperparameter tuning, we combined the training and validation sets to utilize the entire dataset for training.

### 3.2. MultilayerModel

**Figure 2:**
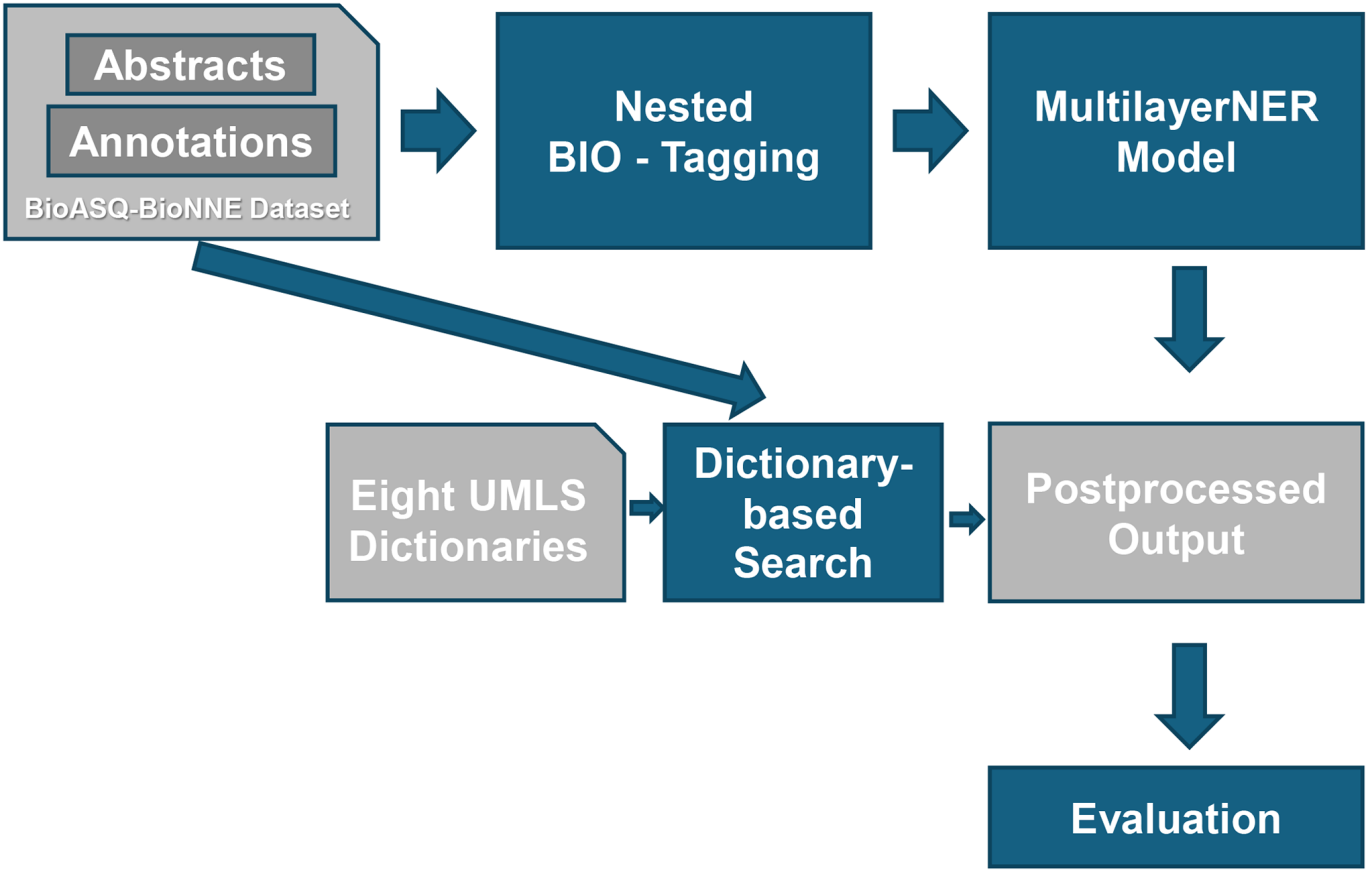
Methodology. BIO-Tagging refers to “Beginning”, “Inside”, and “Outside” tagging.

The core of the method is the MultilayerNERModel, which is our deep learning architecture designed specifically for Nested NER tasks in biomedical texts. This model is built on the robust foundation of the pretrained PubMedBERT, a variant of BERT that has been pretrained on a large corpus of the biomedical literature, making it highly relevant and effective for domain-specific tasks. The overall architecture of our method is shown in Figure 2. The figure represents a comprehensive workflow for recognizing and tagging named entities in biomedical texts. It integrates data from the BioASQ-BioNNE dataset, applies nested BIO-tagging, utilizes a MultilayerNERModel, performs dictionary-based search using UMLS resources, and concludes with postprocessing and evaluation of the results.

#### Base Model:

The base of the model is the pretrained PubMedBERT [10], which provides contextualized word embeddings. We also tried state-of-the-art pretrained BERT and BioBERT as an alternative to PubMedBERT to compare the performance. It can be replaced by any other pretrained model that is compatible with the dataset domain and language.

#### Classification Layers:

A series of six classification layers were added on top of the base model. Each layer was designed to output a specific nested level of NER tags, with each linear layer taking the hidden states from PubMedBERT and mapping them to the required number of labels for that layer. Although the original number of classes in the BioASQ-BioNNE dataset is eight, to support our preprocessed BIO-tagged dataset, the total number of output classes for each classification layer is 17. This includes “B-Class” and “I-Class” for each of the eight original classes, as well as “O” class for the rest of the tokens in the sentences those do not belong to any entity class.

To optimize the performance of our method, we conducted hyperparameter tuning and determined the optimal settings. We utilized the Adam optimizer, known for its efficient handling of sparse gradients and adaptability to different data structures [25]. The hyperparameter settings listed in Table 2 were applied uniformly across all experiments to ensure consistency and facilitate a robust evaluation of our approach.

### 3.3. UMLS Dictionaries

To further enrich the NER process, we leveraged the Unified Medical Language System (UMLS) Metathesaurus 2024AA for vocabulary expansion [26, 27]. We utilized the MRCONSO.RRF data file within UMLS to extract relevant concepts and their child concepts based on the UMLS Semantic Group in Table 3, obtained from the NEREL-BIO GitHub repository (https://github.com/nerel-ds/NEREL-BIO/). This approach allowed us to broaden the model’s ability to recognize entities by incorporating synonyms and related terms. By integrating these expanded vocabularies into our Nested NER system, we aimed to enhance the identification and classification of biomedical entities, ultimately improving the robustness and accuracy of our model.

We extracted the entities from the UMLS dictionaries that match the concept identifiers of our target entity types. These dictionaries served as comprehensive references for the various entities we aimed to identify. Subsequently, we applied these dictionaries to the test data, systematically matching the terms within the text. By capturing the positions of these terms in the test data, we generated an output file that listed the identified entities. We merged this output with the results from our MultilayerNERModel based on BERT, BioBERT, and PubMedBERT. This two-step approach, combining rule-based matching with advanced machine learning techniques, provided a robust mechanism for entity recognition, improving the overall quality and reliability of our extracted data.

### 3.4. Evaluation metrics

We evaluated the model’s performance using precision, recall, F1 score, and macro F1 score with a predefined evaluation script at the competition server of BioASQ. These metrics provide a comprehensive view of the model’s ability to accurately identify and classify named entities in biomedical texts. The equation for precision, recall, F1 score, and macro F1 score are Eq. (1), (2), (3), and (4), respectively.


(1) $$\text{Precision}=\frac{TP}{TP+FP}$$

(2) $$\text{Recall}=\frac{TP}{TP+FN}$$

(3) $$\text{F1}\;\text{Score}=2\times \frac{\text{Precision}\;\times \;\text{Recall}}{\text{Precision}\;+\;\text{Recall}}$$

(4) $$\text{Macro}\;\text{F1}\;\text{Score}=\frac{1}{N}\sum_{i=1}^{N}\text{F1}\;{\text{Score}}_{i}$$

Here, macro F1 score is the average F1 score across the eight entity classes.

## 4. Experimental Results

For this experiment, we employed 6 NVIDIA Tesla V100 GPUs with 32GB of HBM2 VRAM each. The model training and evaluation were implemented using the PyTorch [28] library. To speed up training, the DataParallel class was used to leverage multiple GPUs simultaneously.

**Figure 3:**
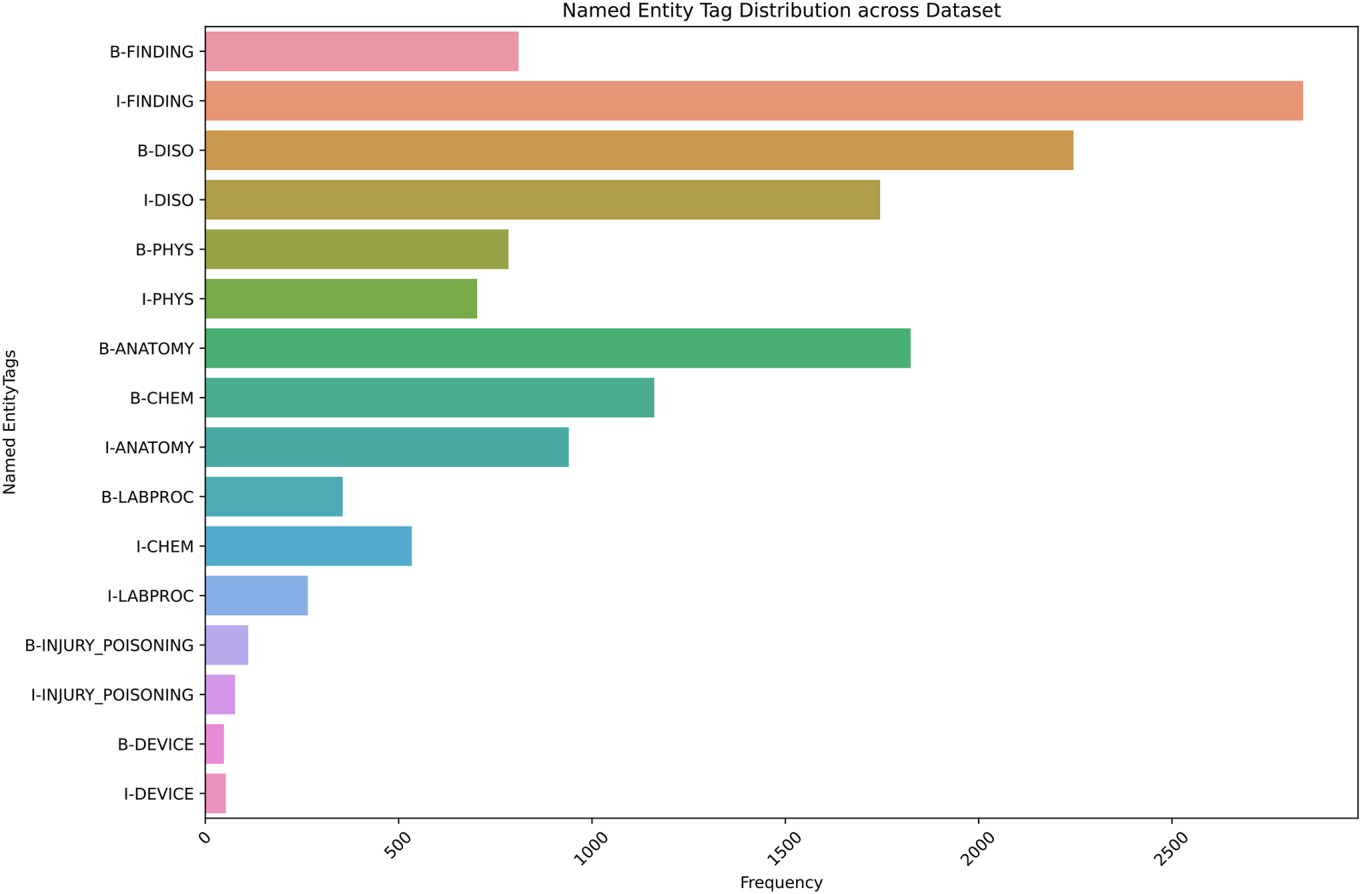
Entity type distribution across the training and development dataset. “B-” and “I-” in the named entity tags represent the BIO-tagging scheme for named entity recognition, with “B” for the beginning of an entity and “I” for subsequent tokens.

The overall distribution of the classes of the dataset after BIO-tagging is illustrated in Figure 3. The bar chart shows the named entity tag distribution, where “I-FINDING” has the highest frequency, while “I-INJURY_ POISONING” and “I-DEVICE” have the lowest frequencies. The frequency of “B-DISO” is higher than “B-ANATOMY”. The distribution of “O” is excluded from the figure as they are not the actual target class of this experiment.

**Figure 4:**
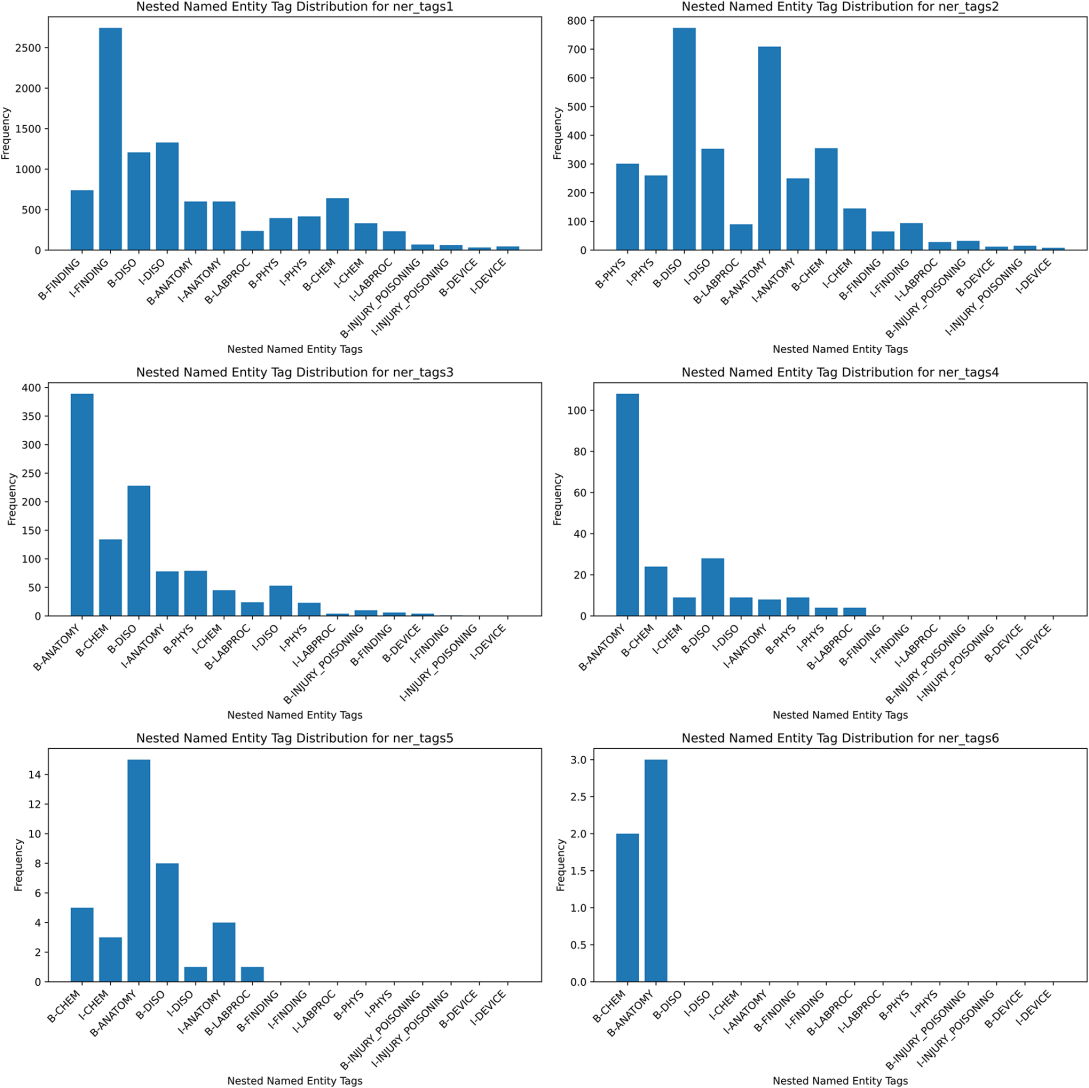
Distribution of nested named entity across the training and development dataset.

Figure 4 provides valuable insights into the behavior and performance of different levels of nested entity tags in recognizing various entity types from the dataset. Overall, the frequency of the “B” (beginning) and “I” (inside) part of “FINDING”, “DISO”, and “ANATOMY” entities are higher across all layers, whereas “DEVICE”, “PHYS”, and “LABPROC” entities show very little or no frequency. The nested entity level 6 hardly had any entity type other than “O”. Again, the frequencies of “O” types are not included in the bar chart as they are not our original target. These distributions highlight the specialized capabilities of certain layers in identifying specific entity types, which can inform the selection of appropriate layers for targeted NER tasks.

We evaluated the performance of various models, namely baseline BERT, BioBERT, PubMedBERT and a combination of UMLS knowledge-based dictionary adaptation. The results are summarized in Table 4, presenting the precision, recall, F1 score, and macro F1 score for each model. PubMedBERT-based MultilayerNERModel achieved the highest overall performance with an F1 score of 67.30% and a macro F1 score of 56.36%. The score demonstrates its effectiveness in capturing the nuances of biomedical texts.

Augmenting the models with UMLS knowledge yielded mixed results. For instance, BioBERT-based MultilayerNERModel, along with UMLS dictionaries, showed an increase in recall (70.97%) compared to BioBERT alone (66.28%), suggesting that UMLS integration helps in better entity recognition coverage. However, this came at the cost of reduced precision (53.58% vs. 64.01%).

PubMedBERT-based MultilayerNERModel, along with UMLS dictionaries, also demonstrated an improvement in terms of highest recall (72.55%) over PubMedBERT-based MultilayerNERModel (68.18%), but similar to BioBERT, it experienced a drop in precision (55.10% vs. 66.45%). Despite this, PubMedBERT-based MultilayerNERModel with UMLS achieved a competitive F1 score of 62.63% and a macro F1 score of 55.46%, indicating that the inclusion of UMLS provides additional benefits in recognizing more entities, albeit with some trade-offs in precision.

We also evaluated our MultilayerNERModel for the Russian and Bilingual Nested NER tracks as well. For the Russian Nested NER track, we used the pretrained SBERT-Large-NLU-RU [29] as the base of our MultilayerNERModel, which is a pretrained BERT-based model specifically tailored for the Russian language. This choice was necessary because BERT, BioBERT, and PubMedBERT are designed for English text only. Our model achieved precision, recall, and F1 scores of 68.59%, 65.34%, and 66.93%, respectively, on the Russian BioNNE dataset. However, the macro F1 score was lower (60.07%) than the F1 score, likely due to potential class imbalance issues.

For the Bilingual Nested NER track, we employed BERT-Base-Multilingual-uncased [30] as the base of our MultilayerNERModel, which is tailored to understand and represent 102 different languages. The precision, recall, and F1 scores were 60.27%, 57.5%, and 58.89%, with a macro F1 score of 50.53%. The performance of our MultilayerNERModel for the Bilingual Nested NER track is lower than the English and Russian Nested NER tracks. Employing a dictionary-based approach may improve the performance of our MultilayerNERModel for Russian and Bilingual tracks.

## 5. Conclusion

Our study demonstrates that the domain-specific models, specifically BioBERT and PubMedBERT-based Nested NER models, significantly outperform the baseline BERT-based Nested NER model in terms of precision, recall, F1 score, and macro F1 score. This improvement underscores the advantage of using models pretrained on biomedical literature for Nested NER tasks within this specialized domain.

Integrating UMLS dictionaries enhances recall, suggesting it helps recognize a broader range of entities. However, the reduction in precision indicates a need for further optimization to balance the trade-offs between precision and recall.

Overall, PubMedBERT stands out as the most effective pretrained model as the base for our MultilayerNERModel, with a promising potential for further enhancement through the strategic incorporation of external knowledge bases like UMLS.

The implications of this research are substantial in the field of biomedical informatics. An improved Nested NER system can facilitate more effective information extraction, aiding researchers in uncovering complex relationships within biomedical literature. This advancement can, in turn, accelerate the discovery of novel insights and advancements in biomedical science. The performance of our MultilayerNERModel emphasizes the capability of multilayer BERT-based architectures in advancing NLP applications in the biomedical domain by addressing the challenges of Nested NER.

## Figures and Tables

**Table 1:** Classwise distribution of the BioASQ-BioNNE 2024 Dataset

Entity type	Train	Dev
DISO	1,200	1,012
ANATOMY	911	897
CHEM	579	575
FINDING	456	348
PHYS	397	379
LABPROC	190	154
INJURY_POISONING	90	20
DEVICE	20	28
**Total**	**3,843**	**3,413**

Refer to Table 3 for details on each entity type.

**Table 2:** Hyperparameter Settings

Hyperparameter	Value
Batch Size	64
Learning Rate	1*e*^−4^
Epochs	40
Maximum Sequence Length	512
Optimizer	Adam

**Table 3:** List of entities and corresponding UMLS semantic groups and concept, obtained from NEREL-BIO GitHub repository.

Entity Type	UMLS Semantic Group	Concept Name	Concept ID	Entity Count (UMLS)	Matched Entities (Abstracts)
ANATOMY	A1.2 Anatomical structure	Body structure	C1268086	586,178	3,063
CHEM	A1.4.1 Chemical	Chemical Substance (organic or inorganic)	C0220806	977,918	784
DEVICE	A1.3.1 Medical device	Medical devices	C0025080	94,190	56
DISO	B2.2.1.2 Pathologic Function	Pathology processes	C0677042	650,482	2,953
FINDING	A2.2 Finding	Experimental finding	C2825141	777,463	1,065
INJURY_POISONING	B2.3 3 Injury and Poisoning	Poisoning/Injury	C0178314	140,659	145
LABPROC	B1.3.1.1 Laboratory Procedure	Medical screening and diagnostic method	C0679541	85,230	450
PHYS	B2.2.1.1 Physiologic Function	Physiological processes	C0031845	168,997	2,288

**Table 4:** Performance Evaluation

Model	Precision (%)	Recall (%)	F1 Score (%)	Macro F1 Score (%)
BERT	53.94	59.32	56.50	44.49
BERT+UMLS Dictionaries	46.39	64.40	53.93	46.15
BioBERT	64.01	66.28	65.12	54.49
BioBERT+UMLS Dictionaries	53.58	70.97	61.06	53.58
PubMedBERT	**66.45**	68.18	**67.30**	**56.36**
PubMedBERT+UMLS Dictionaries	55.10	**72.55**	62.63	55.46

Values in bold indicate the highest performance score achieved amongst the models for each metric
